# PrEP uptake, persistence, and patterns of choice dynamics between oral and long-acting injectable with cabotegravir among sexually and gender-diverse adolescents in Brazil: a multisite cohort implementation study

**DOI:** 10.1016/j.lana.2026.101515

**Published:** 2026-05-28

**Authors:** Laio Magno, Beo Oliveira Leite, Diana Zeballos, Fabiane Soares, Lorenza Dezanet, Mateus Westin, Unaí Tupinambás, Dirceu Greco, Paula Massa, Alexandre Grangeiro, Inês Dourado, PrEP15-19 Choices Study, Thais Regis Aranha Rossi, Priscilla Caires, Filipe Mateus Duarte, Vinícius Damasceno Nascimento, Manuela Cunha Gomes, Márcia Thereza Couto, Luiz Felipe Alves de Sousa, Carolina Cardona Siqueira Lobo, Eliana Miura Zucchi, Eliane Aparecida Sala, Ana Paula Silva, Marília Greco, Patrícia Hafarrany

**Affiliations:** a Instituto Gonçalo Moniz, Fundação Oswaldo Cruz, Salvador, Bahia, Brazil; b Departamento de Ciências da Vida, Universidade do Estado da Bahia, Salvador, Bahia, Brazil; c Instituto de Saúde Coletiva, Universidade Federal da Bahia, Salvador, Bahia, Brazil; d Faculdade de Medicina, Universidade Federal de Minas Gerais, Minas Gerais, Brazil; e Instituto René Rachou, Fundação Oswaldo Cruz, Belo Horizonte, Minas Gerais, Brazil; f Faculdade de Medicina, Universidade de São Paulo, São Paulo, São Paulo, Brazil; g Coordenação Geral de Vigilância em HIV/AIDS, Departamento de HIV/AIDS, Tuberculose, Hepatites Virais e Infecções Sexualmente Transmissíveis, Secretaria de Vigilância em Saúde e Ambiente, Ministério da Saúde, Brasília, Brazil

**Keywords:** PrEP, Choice, Cabotegravir, Sexually and gender diverse populations, Adolescents

## Abstract

**Background:**

Long-acting injectable (LAI) pre-exposure prophylaxis (PrEP) is a newly approved HIV prevention method, and scarce data exist on its real-world implementation among sexually and gender-diverse adolescents (SGDA). This study investigated PrEP uptake, persistence, and patterns of choice dynamics between oral and LAI-PrEP in SGDA in Brazil.

**Methods:**

PrEP15-19 Choices is an implementation study offering both oral and LAI-PrEP with cabotegravir to SGDA aged 15–19, across three Brazilian cities. Data were collected through structured forms and PrEP prescription records (between April 1, 2024, and September 2, 2025). Choice dynamics were defined as the willingness to use each modality of PrEP as well as initiation and switching between injectable and oral options. Descriptive, bivariate, and multiple logistic regression analyses were conducted.

**Findings:**

Of 2150 adolescents reached, 30.0% (n = 644) initiated a PrEP modality: mean age of 18 years old (standard deviation = 1.0), 78.3% were cisgender men who had sex with men and 73.4% self-identified as Black or *Pardo*. Before consultation, the willingness to use daily oral, event-driven (ED), and LAI-PrEP was 32.4%, 17.1%, and 50.5%, respectively, and the actual initiation rates were 38.4% for daily oral PrEP, 18.3% for ED-PrEP, and 43.3% for LAI-PrEP. Among adolescents who started oral PrEP, 24.7% later switched to LAI-PrEP. Conversely, of those who started LAI-PrEP, 10.7% later switched to oral PrEP. Nineteen suicidality events were reported among 13 participants after LAI-PrEP initiation. Participants had higher odds of initiating LAI-PrEP if they had prior experience with oral PrEP (adjusted odds ratio [aOR] 2.07; 95% confidence interval [CI]: 1.35–3.17) or had experienced discrimination based on gender identity or sexual orientation (aOR 1.69; 95% CI: 1.19–2.41). In addition, participants had higher odds to switching from oral to LAI-PrEP if they were Black or *Pardo* (aOR 2.03; 95% CI: 1.03–4.02). The persistence rates among oral and LAI-PrEP users were 79.3% and 88.8%, respectively.

**Interpretation:**

Adolescent-centered HIV prevention should prioritize choice and psychosocial support. The preference for long-acting formulations among adolescents experiencing discrimination and among Black and *Pardo* individuals highlights their potential to reduce structural barriers and advance equity in PrEP access.

**Funding:**

UNITAID and Brazilian Ministry of Health.

**Research in context**

**Evidence before this study**

We searched PubMed for articles reporting studies on pre-exposure prophylaxis (PrEP) choice or initiation. The following search terms were used: (cabotegravir) AND (uptake∗ OR implement∗ OR choice∗ OR initiation∗) AND (pre-exposure prophylaxis OR PrEP). Our search was limited to studies published before May 9, 2025, without language limitations. We also included studies in the revision stage between May 9, 2025, and December 10, 2025. We identified five studies reporting on PrEP choice or initiation: three conducted in Africa (Zimbabwe, Kenya, Uganda, and South Africa) and two in the United States. One intervention study that offered participants dynamic choices between long-acting injectable PrEP (LAI-PrEP) with cabotegravir, oral PrEP, and post-exposure prophylaxis reported that 52.0% initially chose LAI-PrEP; by week 48, 56.6% had received at least one LAI-PrEP injection. The HPTN 084-01 trial, a single-arm, open-label Phase 2 study among cisgender adolescent women, found that 92.4% opted to continue LAI-PrEP after trial completion. The PrEPared to Choose study assessed PrEP method selection from pregnant and lactating women in the baseline, 82.7% initiated LAI-PrEP. In a US-based syringe services program, 88.0% of cisgender women who injected drugs chose LAI-PrEP. Another early implementation study at a Safety Net Hospital-based Primary Care Center in the southern United States showed that 56.6% of the 221 referred individuals accepted LAI-PrEP, with 34.8% initiating LAI-PrEP. However, the latter two studies did not explicitly state whether participants were offered multiple PrEP options. Previous research among Brazilian adolescents and young adults found that 81.5% expressed willingness to use long-acting injectable PrEP. Although early evidence suggests high acceptability and preference, robust real-world data on LAI-PrEP uptake and choice among adolescents are limited, primarily because of the lack of data beyond clinical trial settings.

**Added value of this study**

This is the first real-world implementation study globally to report uptake and patterns of choice dynamics across oral and LAI PrEP among sexually and gender-diverse adolescents (SGDA). The overall PrEP uptake was 30.0%, comparing favorably with the earlier oral-only PrEP15-19 cohort. Before clinical consultation, the willingness to use PrEP was diverse: 50.5% reported preference for LAI-PrEP, 32.4% for daily oral PrEP, and 17.1% for event-driven PrEP. Despite this, actual initiation was lower for LAI PrEP (43.3%), underscoring a gap between expressed willingness and real-clinical uptake. We document an intention–initiation gap, quantify willingness→initiation→switching pathways, and identify associated factors related to initiation and switching. Adolescents with prior oral PrEP experience and those reporting recent discrimination based on gender identity or sexual orientation were more likely to initiate LAI-PrEP. Switching occurred more often from oral to LAI-PrEP than vice versa, with Black and Pardo adolescents showing higher odds of switching to LAI PrEP. In our cohort, persistence was higher with LAI-PrEP than with oral PrEP. Importantly, we also reported 19 suicidality events after LAI-PrEP initiation, most either accompanied by worsening in patient health questionnaire (PHQ-9) scores or occurring in adolescents with moderate-to-severe baseline scores. Collectively, these findings provide evidence to guide adolescent-centered PrEP programs that expand modality choices while addressing discrimination and structural inequities.

**Implications of all the available evidence**

Our findings highlight the critical importance of offering a diverse range of HIV prevention strategies that are responsive to adolescents’ specific needs and preferences. Long-acting formulations may play a key role in addressing the effects of discrimination based on gender identity and sexual orientation, and racism in the Brazilian context, which often deters marginalized adolescents from accessing healthcare services. This underscores the need to create stigma-free and affirming environments where young people feel safe and empowered to engage in HIV prevention. These findings call for integrated care models that combine mental health support with HIV prevention services, particularly for those experiencing multiple layers of social vulnerability. To fully realize the potential of LAI-PrEP, implementation strategies must focus on addressing adolescent concerns regarding injectable formulations, enhancing clinical eligibility assessments, and increasing awareness of the unique benefits of long-acting options. Expanding and diversifying PrEP modalities while actively combating discrimination and supporting adolescent mental health can offer a promising path for improving adherence and advancing HIV prevention outcomes among SGDA.

## Introduction

The HIV epidemic has disproportionately affected adolescent populations worldwide.^1^ In several African countries, adolescent girls represent the most affected group,^1^ whereas in many countries across the Americas, adolescent boys exhibit higher HIV incidence rates.^2^^,^^3^

Brazil, which has one of the highest HIV burdens in Latin America,^4^ has seen rising HIV and AIDS incidences among adolescents and young men in the general population.^2^ Although the national surveillance data do not disaggregate the most affected subpopulations, research has identified a high HIV burden among sexually- and gender-diverse adolescents (SGDA).^3^^,^^5^

Within this context, studies have reported a decline in condom use among SGDA,^6^ as well as persistent barriers to accessing HIV prevention services and technologies in Brazil.^7^ In response, the Brazilian Ministry of Health has expanded access to oral pre-exposure prophylaxis (PrEP) in 2022 to include adolescents aged 15–17 years.^8^ Despite this advancement, demonstration studies continue to highlight challenges related to both the adherence and persistence of oral PrEP use among these populations.^9^, ^10^, ^11^ Moreover, adolescents account for only 0.2% of all current PrEP users in Brazil (n = 140,412),^12^ although Brazil has around 14 million adolescents aged 15–19 years.^13^

Long-acting injectable PrEP (LAI-PrEP), specifically cabotegravir (CAB), has shown superior efficacy in preventing HIV compared to oral formulations^14^^,^^15^ and may offer a more effective alternative for improving adherence among SGDA. However, real-world demonstration studies evaluating the use of LAI-PrEP with CAB in this population are lacking, especially in the context of increasing options for other PrEP modalities and emerging patterns of use. We define choice dynamics as the continuum that begins with the willingness to use a given modality, shaped by demand creation strategies (DCS), product availability, and marketing, that progresses through initiation, following shared decision-making with health providers, and extends to subsequent switching between modalities. Thus, this study investigated PrEP uptake, persistence, and patterns of choice dynamics between oral and LAI-PrEP among SGDA in Brazil.

## Methods

### Study design

PrEP15-19 Choices is a multisite cohort implementation study guided by the Reach, Effectiveness, Adoption, Implementation, and Maintenance framework.^16^ This study was conducted in three cities in Brazil. In Salvador, the PrEP service was located in the *Casarão da Diversidade*, a center focused on SGDA rights. In Belo Horizonte, the PrEP service operates within the Youth Reference Center. In São Paulo, PrEP services were delivered at two distinct sites: a community-based clinic led by nurses and peer educators in partnership with CASA 1, an NGO that offers shelter, cultural programming, and health services for sexual and gender diverse populations; and a primary care clinic of the Brazilian National Health System (*SUS*), where PrEP is provided by physicians. This longitudinal study with an initial cross-sectional wave included participants recruited, enrolled, and followed up between April 1, 2024, and September 2, 2025.

### Ethical statement

The study was approved by the World Health Organization (WHO) Ethics Review Committee (ERC) (May 29, 2023; ERC 0003789) and by the local ERC from the universities coordinating the study: the University of São Paulo (May 26, 2023; #CAAE 67076822.6.1001.0068), the Federal University of Bahia (August 4, 2023; #CAAE 67076822.6.2002.5030), and the Federal University of Minas Gerais (September 11, 2023; #CAAE 67076822.6.2001.5149). Adolescents aged >18 years signed a consent form, and those aged 15–17 years signed an informed assent form, waiving the need for the approval of a legal guardian, except in cases where it was found that the adolescent did not have the necessary autonomy, according to a specialized psychological assessment, or mental and/or intellectual impairment that does not allow PrEP use. The parental consent form was waived by local ERCs based on our previous implementation of a daily oral PrEP study with adolescents. The trial registration number is #RBR-104736f4. The detailed protocol procedures have been described by Dourado et al.^17^

### Participants

This study included SGDA participants aged 15–19 years who self-identified as cisgender men who have sex with men (MSM), transgender women (TGW), transgender men (TGM), or non-binary (NB) individuals assigned male at birth. Eligible participants had an elevated vulnerability or risk of HIV infection, including behaviors such as condomless anal sex, history of sexually transmitted infections (STIs), or prior use of post-exposure prophylaxis (PEP) and PrEP.

General exclusion criteria were: confirmed HIV-positive status at screening or enrollment; body weight below 35 kg; evidence of mental and/or intellectual impairment that compromised the ability to make an informed decision to initiate PrEP, as determined by a psychological assessment; and participation in another interventional trial involving PrEP agents, experimental medication, and/or HIV vaccines. Oral PrEP-specific exclusion criteria included renal dysfunction (glomerular filtration rate <60 mL/min/1.73 m^2^) and history of spontaneous bone fractures.

Exclusion criteria specific to LAI-PrEP included: surgically implanted buttock implants or fillers; diagnosis of chronic hepatitis B requiring antiviral therapy; current or chronic liver disease or known hepatic/biliary abnormalities; any coagulopathy contraindicating intramuscular injections; use of medications such as carbamazepine, oxcarbazepine, phenobarbital, phenytoin, rifampin, or rifapentine; planned relocation from the study site during the study period; and reported hepatic dysfunction. Screening was deferred for participants reporting liver disease until liver function test results were available. Individuals with alanine aminotransferase (ALT) levels ≥5 times the upper limit of normal (ULN), or ≥3 × ULN with total bilirubin ≥1.5 × ULN, were excluded. Individuals with a known allergy to cabotegravir or its components were also excluded. For TGM, additional exclusion criteria were: a positive pregnancy test at enrollment; current breastfeeding; and intention to become pregnant.

During follow-up, new exclusion criteria were introduced in phases after cases of suicidality were identified, reviewed, and reported by the Clinical Medical Committee. From February 1 to March 31, 2025, individuals already receiving LAI-PrEP and new participants were excluded if they had i) a patient health questionnaire (PHQ-9) score ≥20 or ii) PHQ-9 score ≥15 accompanied by at least one of the following: history of psychiatric hospitalization; documented absence from school or work owing to a psychiatric diagnosis; use of hallucinogenic substances (e.g., LSD, ecstasy, mushrooms, mescaline, or peyote; marijuana excluded); or concurrent use of two or more mental-health medications (e.g., anxiolytics, antidepressants, antipsychotics, lithium, methylphenidate, or other amphetamines). From April 1, 2025 onward these criteria were changed, and individuals were excluded from LAI-PrEP if they met at least one of the following criteria: i) any prior suicide attempts, or ii) self-harm in the past 6 months that led to care at a healthcare facility. This alteration of suicide risk screening criteria was intended to increase specificity with minimal loss of sensitivity, focusing on direct indicators of suicidality rather than broader markers of psychiatric vulnerability.^18^

All suicidality events were systematically reported to the Institutional Review Boards (IRBs). Upon identification or disclosure of an event, participants were provided with immediate and comprehensive support. Care delivery was facilitated by a multidisciplinary team—comprising a physician, a nurse, a psychologist, and a social worker—supplemented by peer educators. These educators played a crucial role in maintaining communication between scheduled visits and reinforcing consistent engagement with the service. Where appropriate, participants were referred to mental health services of the national health system. Furthermore, the project ensured timely access to consultations with an adolescent psychiatrist for cases requiring immediate specialized evaluation.

### Procedures

Recruitment was conducted both virtually and in person through demand creation strategies (DCS), implemented in collaboration with community-based organizations and PrEP service sites. The DCS included peer educators who were members of sexually and gender-diverse youth communities. They were involved in leading activities including discussions on sexual orientation, gender identity, sexual behavior, and HIV prevention, as well as the distribution of HIV self-tests, condoms, and lubricants in venues and other youth social spaces. For online recruitment, peer educators use social media and dating applications (apps) to promote the project. Simultaneously, prevention supplies were either mailed to participants or made available for collection at PrEP services, according to their preferences.

All SGDA reached through DCS received detailed information about the study and were invited to attend the PrEP services. At these services, participants were screened, and all eligible individuals were offered the opportunity to choose one of the PrEP options during the consultation: oral PrEP, consisting of tenofovir disoproxil fumarate/emtricitabine (TDF/FTC), provided either as a daily or an event-driven regimen (2 pills taken 2–24 h before sexual intercourse, followed by 1 pill 24 h after the initial dose and another pill 48 h after the initial dose, i.e., 2 + 1 + 1), or the LAI-PrEP, consisting of intramuscular cabotegravir (CAB) 600 mg (3 mL).

For oral PrEP, participants were screened for HIV using a fourth-generation HIV rapid test at all visits. For LAI-PrEP, during enrollment and at each visit for injection restart or discontinuation, individuals were tested using a point-of-care qualitative nucleic acid test for HIV (GeneXpert HIV-1). Subsequent injections were administered based on a negative fourth-generation HIV rapid test performed at each visit.

Participants could initiate either PrEP modality on the same day and were prospectively followed up at 1 month in both arms, every 4 months thereafter in the oral PrEP arm, and every 2 months thereafter in the LAI-PrEP arm. The study allowed participants to switch between PrEP modalities over time.

At all PrEP initiation and follow-up visits, the participants received counseling on HIV risk reduction. Additionally, participants were tested for STIs, safety monitoring (liver and renal function tests), adverse events, sexual risk compensation, access to differentiated services, and other HIV-prevention tools.

PrEP initiation by modality was guided by a shared decision-making process that considers each participant’s individual needs, preferences, and sexual behaviors. Socio-behavioral and clinical questionnaires addressing PrEP use, additional preventive practices, and sexual behavior were administered by trained researchers at enrollment and four-month intervals during follow-up visits. To support engagement, retention, and adherence, the participants received both virtual and in-person assistance from peer navigators and psychosocial care professionals. All consultations were documented in electronic medical records by attending healthcare professionals.

Data sources included sociobehavioral questionnaires and prescription records. PrEP uptake was defined as the proportion of participants who initiated PrEP in any modality divided by the total number of individuals reached through DCS. Subsequent analyses were restricted to participants who initiated PrEP. Patterns of PrEP choice were analyzed as follows: i) willingness to use a PrEP modality was defined by intention to use one of the three modalities (i.e., LAI-PrEP, daily oral PrEP, or event-driven PrEP), as reported in the questionnaire prior to clinical consultation; ii) PrEP initiation was defined as the first modality prescribed, categorized as LAI-PrEP or oral PrEP (i.e., composed of daily and event-driven regimens), following shared decision-making with a health professional; iii) switching was defined as the first change in PrEP modality after initiation during the study period (i.e., from LAI-PrEP to oral PrEP or vice versa). Switching was assessed by identifying whether the participants remained in their initial modality or switched during the follow-up period from study entry until the end of the observation period (September 2, 2025). Additionally, persistence was defined as continued PrEP use, with no interval greater than 90 days since the last scheduled visit for oral PrEP users and no interval greater than 60 days for injectable PrEP users, assessed at the study censoring date (September 2, 2025).

All covariates analyzed were collected at the enrollment and were: Demographics: age (15–17 vs. 18–19), population (MSM vs. TGW vs. TGM vs. NB), study site (Salvador vs. Belo Horizonte vs. São Paulo), self-reported race/skin color (White vs. Black or *Pardo*—i.e., the last one is a mixed-race, according to the Brazilian Institute of Geography and Statistics classification for race, which is considered together as a unique Black group in Brazil), schooling (elementary/high school vs. higher education), income (none/cash transfer program/less than 1 minimum wage vs. 1–2 vs. more than 2 minimum wages), housing (living with vs. without parents or relatives), private health insurance (yes vs. no); Sexual behavioral: self-reported sexual orientation (heterosexual vs. homosexual vs. pansexual/bisexual), relationship status (not in a relationship vs. in a relationship), timeframe of sexual engagement (had been sexually active less than 1 year vs. 1 year or more), number of partners in the past 3 months (0–1 vs. 2+), condomless anal sex in the past 6 months (no vs. yes), transactional sex in the past 3 months (no vs. yes), forced sex history (no vs. yes), STI in the past 12 months (no vs. yes), HIV risk self-perception (high/moderate vs. low/none), prior oral PrEP use (no vs. yes); Psychosocial: experiences of physical assault (self-reported physical violence because of the participant’s sexual orientation or gender identity) within the past 6 months (no vs. yes) and discrimination based on gender identity or sexual orientation in past 6 months (no vs. yes); PHQ-9 depression score (none vs. mild/moderate vs. moderately severe/severe) at baseline and first assessment after any suicide attempt.

### Statistical analysis

Descriptive analyses were performed using absolute and relative frequencies for categorical variables and means with standard deviation (SD) for continuous variables in the total sample. Missing values were excluded from the analysis. Bivariate associations between PrEP initiation, switching to LAI-PrEP, and covariates were assessed using Pearson’s chi-squared test or Fisher’s exact test, as appropriate. Variables with a p-value < 0.20 in the bivariate analysis, and age, skin color, and population group were included in the multivariable models. Mixed-effects binomial logistic regression was used to estimate adjusted odds ratios (aORs) and 95% confidence intervals (95% CI) for PrEP initiation and switching to LAI-PrEP, and the study site was included as a random intercept to account for unobserved differences across cities. The final models were defined using a non-automated backward selection strategy, retaining variables based on theoretical relevance, and the evaluation of the Akaike information criterion (AIC), Bayesian information criterion (BIC), and likelihood-ratio test to identify the most parsimonious model. Because of the introduction of mental health-related eligibility criteria for LAI-PrEP initiation and switching, we conducted a sensitivity analysis estimating two models: model 1, excluding participants who expressed willingness to use LAI-PrEP but were ineligible; and model 2, classifying participants who were ineligible for LAI-PrEP based on mental health criteria as LAI-PrEP users if they mentioned willingness to use LAI-PrEP (Supplementary Table S3). All analyses were performed using R software version 4.4.1.^19^

### Role of the funding source

The funders had no role in the study design, data collection, data analysis, data interpretation, or writing of the report.

## Results

**Fig. 1:**
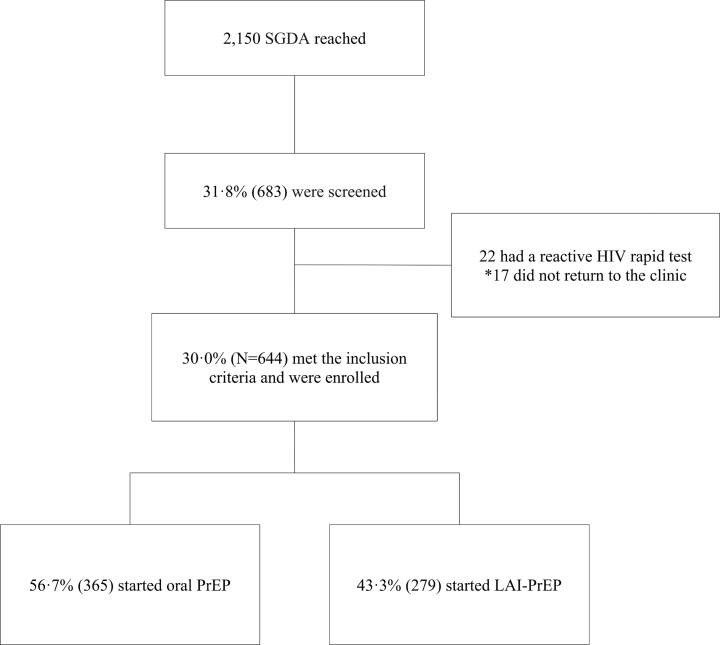
Flowchart of SGDA reached, screened, and initiated on PrEP, April/2024–September/2025, PrEP15-19 Choice study. ∗ PrEP was not initiated. SGDA: sexually and gender-diverse adolescents; PrEP: pre-exposure prophylaxis; LAI-PrEP: long-acting injectable pre-exposure prophylaxis.

Of the 2150 adolescents recruited using DCS, 644 (uptake of 30.0%) were enrolled in the PrEP modality (Fig. 1). Participants who started PrEP differed from those reached by DCS in having a lower proportion of MSM and White individuals (Supplementary Table S1). Among the enrolled participants, the majority were aged 18–19 years (74.7% [481/644]) and mean age of 18 years old (SD = 1.0), identified as MSM (78.3% [504/644]), and were from São Paulo site (43.0% [277/644]). Regarding self-reported sexual orientation, 59.6% (382/641) identified as homosexual, 26.8% (172/641) as bisexual, 6.2% (40/641) pansexual and 7.3% (47/641) as heterosexual. Most participants identified as Black or *Pardo* (73.4% [471/642]), had completed or were currently enrolled in elementary education or high school (69.5% [445/640]), reported a household income of more than two minimum wages (∼US$251.00) (49.5% [276/557]), and lived with their parents or relatives (83.2% [494/594]). Additionally, 31.5% (186/591) of the participants reported having private health insurance (Table 1).

**Table 1:** Sociobehavioral characteristics of SGDA enrolled, April/2024–September/2025, PrEP15-19 Choices Study.

Variables	Total (N = 644)		PrEP initiation (N = 644)				p-value	Switched to LAI-PrEP (N = 365)				p-value
			Oral PrEP^d^		LAI-PrEP			No		Yes		
	n	%^c^	n	%	n	%		n	%	n	%	
Age							0.51^a^					0.36^a^
15–17 years old	163	25.3	96	26.3	67	24.0		69	25.1	27	30.0	
18–19 years old	481	74.7	269	73.7	212	76.0		206	74.9	63	70.0	
Population							0.67^b^					0.56^b^
MSM	504	78.3	292	80.0	212	76.0		217	78.9	75	83.3	
TGW	78	12.1	40	11.0	38	13.6		32	11.6	8	8.9	
TGM	9	1.4	5	1.4	4	1.4		3	1.1	2	2.2	
NB	53	8.2	28	7.7	25	9.0		23	8.4	5	5.6	
Site							<0.001^a^					0.98^a^
Salvador	231	35.9	160	43.8	71	25.4		120	43.6	40	44.4	
Belo Horizonte	136	21.1	64	17.5	72	25.8		48	17.5	16	17.8	
São Paulo	277	43.0	141	38.6	136	48.7		107	38.9	34	37.8	
Sexual orientation							0.09^a^					0.47^b^
Heterosexual	47	7.3	30	8.3	17	6.1		25	9.1	5	5.7	
Homosexual	382	59.6	220	60.8	162	58.1		163	59.3	57	65.5	
Bisexual	172	26.8	93	26.7	79	28.3		72	26.2	21	24.1	
Pansexual	40	6.2	19	5.3	21	7.5		15	5.5	4	4.6	
Race/skin color							0.78^a^					0.05^a^
White	171	26.6	94	25.8	77	27.8		78	28.4	16	17.8	
*Pardo*	224	34.9	131	35.9	93	33.6		91	33.1	40	44.4	
Black	247	38.5	140	38.4	107	38.6		106	38.5	34	37.8	
Schooling							0.25^a^					0.04^a^
Elementary school/High school	445	69.5	259	71.3	186	67.1		188	68.6	71	79.8	
Higher education	195	30.5	104	28.7	91	32.8		86	31.4	18	20.2	
Income							0.10^a^					0.18^a^
No income or on a cash transfer program/Less than 1 minimum wage	65	11.7	42	13.2	23	9.6		36	15.1	6	7.6	
1–2 minimum wage	216	38.8	126	39.7	90	37.5		90	37.8	36	45.6	
More than 2 minimum wages	276	49.5	149	47.0	127	52.9		112	47.1	37	46.8	
Housing							0.50^a^					0.06^a^
Living with parents or relatives	494	83.2	285	84.1	209	82.0		209	82.0	76	90.5	
Not living with parents or relatives	100	16.8	54	15.9	46	18.0		46	18.0	8	9.5	
Private health insurance							0.25^a^					0.06^a^
No	405	68.5	238	70.4	167	66.0		181	71.3	58	68.2	
Yes	186	31.5	100	29.6	86	34.0		73	28.7	27	31.8	
PHQ-9							0.06^a^					0.01^a^
None	94	16.8	50	15.3	44	18.7		42	17.1	8	9.9	
Mild and moderate	358	63.8	204	62.6	154	65.5		142	58.0	62	76.5	
Moderately severe and severe	109	19.4	72	22.1	37	15.7		61	24.9	11	13.6	
Relationship status							0.10^a^					0.39^a^
Not in a relationship	416	65.4	246	68.1	170	61.8		188	69.4	58	64.4	
In a relationship	220	34.6	115	31.9	105	38.2		83	30.6	32	35.6	
Timeframe of sexual engagement							0.13^a^					0.51^a^
Less than 1 year	170	29.8	104	32.4	66	26.5		77	31.4	27	35.5	
1 year or more	400	70.2	217	67.6	183	73.5		168	68.6	49	64.5	
Number of sexual partners in the past 3 months							0.21^a^					0.73^a^
0–1	204	32.0	123	34.1	81	29.3		91	33.6	32	35.6	
2 or more	433	68.0	238	65.9	195	70.7		180	66.4	58	64.4	
Condomless anal sex in the past 6 months							0.45^a^					0.19^a^
No	117	18.2	70	19.2	41	15.0		57	20.7	13	14.4	
Yes	527	81.8	295	80.8	232	85.0		218	79.3	77	85.6	
Transactional sex in the past 3 months							0.21^a^					0.79^b^
No	593	93.4	341	94.5	252	92.0		255	94.1	86	95.6	
Yes	42	6.6	20	5.5	22	8.0		16	5.9	4	4.4	
Forced sex history							0.56^a^					0.74^a^
No	406	68.4	235	69.3	171	67.1		178	69.8	57	67.9	
Yes	188	31.6	104	30.7	84	32.9		77	30.2	27	32.1	
STI in the last 12 months							0.30^a^					0.85^a^
No	473	80.2	276	81.7	197	78.2		208	81.9	68	80.9	
Yes	117	19.8	62	18.3	55	21.8		46	18.1	16	19.0	
HIV risk self-perception							0.87^a^					0.12^a^
High/moderate	150	23.6	86	23.8	64	23.3		70	25.8	16	17.8	
Low/None	486	76.4	275	76.2	211	76.7		201	74.2	74	82.2	
Prior oral PrEP use							<0.001^a^					0.05^a^
No	467	72.5	287	78.6	180	64.5		223	81.1	64	71.1	
Yes	177	27.5	78	21.4	99	35.5		52	18.9	26	28.9	
Discrimination based on gender or sexual orientation in the last 6 months							0.003^a^					0.65^a^
No	346	54.6	215	59.7	131	47.8		160	59.0	55	61.8	
Yes	288	45.4	145	40.3	143	52.2		111	41.0	34	38.2	
Experiences of physical assault within the past 6 months							0.70^a^					0.08^a^
No	606	95.3	345	95.6	261	94.9		262	96.7	83	92.2	
Yes	30	4.7	16	4.4	14	5.1		9	3.3	7	7.8	

SGDA, sexually and gender-diverse adolescents; PrEP, pre-exposure prophylaxis; MSM, men who have sex with men; TGW, transgender women; TGM, transgender men; NB, non-binary; PHQ-9, Patient Health Questionnaire-9.

a Pearson’s chi-squared.

b Fisher’s exact test.

c Percentages were calculated using non-missing responses only.

d Oral PrEP refers to both daily and on-demand regimens, which were combined into a single category for this analysis.

Regarding mental health, 63.8% (358/561) of the participants scored in the mild to moderate range on the PHQ-9 at baseline (Table 1). Nineteen suicidality events were reported among 13 participants after LAI-PrEP initiation, occurring between 22 and 270 days of follow-up. Most cases involved cisgender men and transgender women, with a higher frequency in Black and *Pardo* individuals. Five of the participants experienced recurrent episodes. Although a causal relationship could not be established, many events were accompanied by worsening PHQ-9 scores between baseline and the first post-event assessment (Table 2).

**Table 2:** Characteristics of suicidality cases after LAI-PrEP initiation among SGDA, PrEP15-19 Choices Study, April/2024–September/2025.

ID^a^	Study site	Onset time of AE (Month/Year)	PHQ-9 before AE	First PHQ-9 after AE	Age^b^	Gender identity	Race/skin color
1	Salvador	05/2024	Moderate	Severe	18	Cisgender man	Black
2	São Paulo	06/2024	Mild	Moderately severe	18	Transgender woman	White
3	Belo Horizonte	06/2024	Moderate	Mild	16	Transgender woman	Black
4	São Paulo	06/2024	None	Severe	20	Non-binary assigned male at birth	Black
3	Belo Horizonte	08/2024	Mild	Moderate	16	Transgender woman	Black
5	São Paulo	09/2024	Severe	Severe	19	Transgender woman	White
3	Belo Horizonte	10/2024	Mild	Moderate	16	Transgender woman	Black
6	Belo Horizonte	10/2024	Mild	Moderate	20	Cisgender man	*Pardo*
7	São Paulo	11/2024	Mild	Moderately severe	20	Transgender woman	*Pardo*
8	Belo Horizonte	11/2024	Moderate	Moderate	17	Cisgender man	*Pardo*
9	Belo Horizonte	01/2025	Moderate	Severe	18	Cisgender man	*Pardo*
5	São Paulo	01/2025	Severe	Severe	19	Transgender woman	White
10	São Paulo	01/2025	Severe	Mild	20	Cisgender man	*Pardo*
11	Belo Horizonte	01/2025	Mild	Severe	20	Transgender woman	Black
11	Belo Horizonte	02/2025	Mild	Severe	20	Transgender woman	Black
6	Belo Horizonte	02/2025	Moderate	None	20	Cisgender man	*Pardo*
12	São Paulo	02/2025	Mild	Mild	18	Cisgender man	Black
13	Salvador	06/2025	Moderate	Severe	20	Cisgender man	*Pardo*
13	Salvador	06/2025	Moderate	Severe	20	Cisgender man	*Pardo*

AE, adverse event; ID, number of identifications; LAI-PrEP, Long-acting injectable preexposure prophylaxis; SGDA, sexually and gender-diverse adolescents; PHQ-9, Patient Health Questionnaire-9.

a Thirteen participants experienced at least one suicidality event. Each line represents a separate event.

b Age refers to age at the first PHQ-9 after the AE, not age at enrollment; therefore, some participants were older than 19 years at the event despite meeting eligibility criteria at study entry.

Most participants were not in a relationship (65.4% [416/636]); 70.2% (400/570) had been sexually active for 1 year or more, and 68.0% (433/637) reported having two or more sexual partners in the past three months. Condomless anal sex in the previous 6 months was reported by 81.8% (527/644) of the participants. Transactional sex was reported by 6.6% (42/635), and 31.6% (188/594) reported having experienced forced sex at some point in their lives. Experiences of discrimination based on gender identity or sexual orientation in the past 6 months were reported by 45.4% (288/634), while 4.7% (30/636) reported experiencing physical assault in the past 6 months. STIs within the past 12 months were reported by 19.8% (117/590). Although 76.4% (486/636) perceived themselves to be at low or none risk of HIV, 72.5% (467/644) of the participants enrolled in PrEP were participants without prior PrEP use (Table 1).

**Fig. 2:**
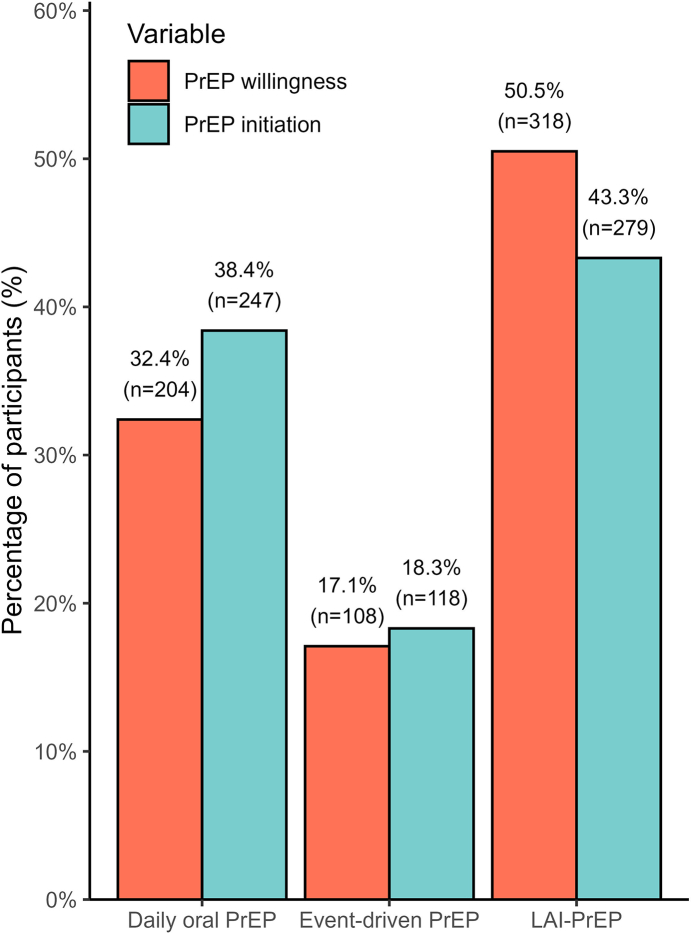
Percentages of willingness to use PrEP vs. actual initiation, April/2024–September/2025, PrEP15-19 Choice study. PrEP, pre-exposure prophylaxis; LAI-PrEP, long-acting injectable pre-exposure prophylaxis. Percentages were calculated using non-missing responses only (n = 630 for PrEP willingness; n = 644 for PrEP initiation).

Regarding the initial choice of the enrolled participants in PrEP, 49.5% (312/630) expressed willingness to initiate oral PrEP—32.4% (204/630) daily regimen and 17.1% (108/630) event-driven regimen—whereas 50.5% (318/630) preferred LAI-PrEP. After clinical consultation, 56.7% (365/644) ultimately initiated oral PrEP — 38.4% (247/644) daily and 18.3% (118/644) event-driven — whereas 43.3% (279/644) initiated LAI-PrEP (Fig. 2).

Participants choosing LAI-PrEP primarily valued not having to worry about forgetting to take daily pills (79.8% [198/248]) and expressed a preference for this modality over daily oral pills (77.4% [192/248]). The most common reasons for choosing oral PrEP differed by regimen. Those opting for daily oral PrEP cited the unpredictability of sexual activity (56.3% [112/199]) and greater confidence in the daily regimen (48.7% [97/199]), whereas participants choosing event-driven PrEP emphasized having less frequent sexual encounters (68.6% [81/118]) and using less medication (48.3% [57/118]) (Table 3).

**Table 3:** Reasons for PrEP initiation by modality, April/2024 to September/2025, PrEP15-19 choices.

Reasons for choosing LAI-PrEP^b^	Total (N = 248)	
	n	%^a^
I don’t have to worry about forgetting to take pills.	198	79.8
It’s better than taking medication daily.	192	77.4
It’s better than taking medication every time I have sex.	131	52.8
I don’t have to carry medication with me.	119	48.0
An injection every 2 months prevents HIV.	82	33.1
I don’t have to worry about HIV for 2 months.	80	32.3
No one will see that I’m using PrEP pills.	57	23.0
It may have fewer side effects.	45	18.1

Reasons for choosing daily oral PrEP^b^	Total (N = 199)	
	n	%^a^
I do not know in advance when I will have sexual intercourse.	112	56.3
I have greater trust in daily PrEP.	97	48.7
I find it difficult to remember the correct way to use event-driven PrEP (2 + 1 + 1) before and after sex.	89	44.7
I have frequent sexual activity.	29	14.6

Reasons for choosing event-driven PrEP^b^	Total (N = 118)	
	n	%^a^
I do not have frequent sexual activity.	81	68.6
It is better than taking medication daily.	57	48.3
I have a predictable sex life; I always know when I will have sex.	55	46.6
I have difficulty remembering to take medication every day.	31	26.3
It may cause fewer side effects.	11	9.3

PrEP, pre-exposure prophylaxis; LAI-PrEP, long-acting injectable pre-exposure prophylaxis.

a Percentages were calculated using non-missing responses only.

b Participants could select more than one reason.

**Fig. 3:**
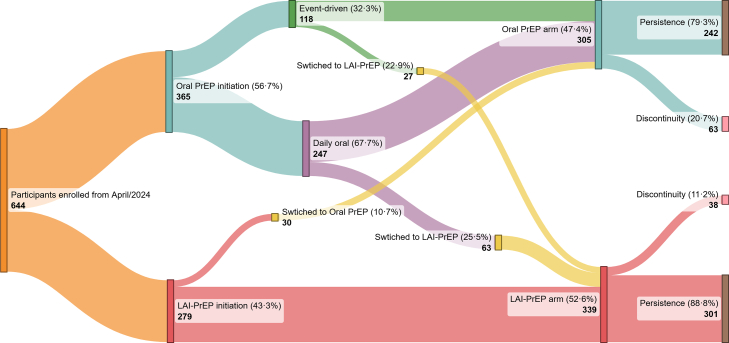
Patterns of PrEP initiation and switching among SGDA, April/2024 to September/2025, PrEP15-19 Choices Study. McNemar’s chi-squared test p-value < 0.001 for switching from event-driven (22.9%) or daily oral (25.5%) PrEP to LAI-PrEP which were higher than switching from LAI-PrEP (10.7%) to daily oral PrEP; Fisher’s exact test p-value < 0.001 for differences between persistence in oral PrEP arm vs. LAI-PrEP arm (79.3 vs. 88.8%); all switches from LAI-PrEP to Oral PrEP were on daily modality. SGDA: sexually and gender-diverse adolescents; PrEP: pre-exposure prophylaxis; LAI-PrEP: long-acting injectable pre-exposure prophylaxis.

Among adolescents who initiated oral PrEP, 24.7% (90/365) switched to LAI-PrEP (22.9% [27/118] from event-driven and 25.5% [63/247] from daily oral PrEP) during follow-up. In contrast, among those who started with LAI-PrEP (43.3% [279/644]), 10.7% (30/279) switched to the oral regimen during follow-up, significantly lower than the rate of switching to LAI-PrEP (p < 0.001) (Fig. 3). The main reasons for switching from LAI-PrEP to oral PrEP were mental health concerns or an increased risk of mental health issues (n = 12), pain or pyrexia at the injection site (n = 9), and their own decision with no reason given (n = 5) (Supplementary Table S2). After these switches, the distribution of PrEP was 52.6% (339/644) for LAI-PrEP and 47.4% (305/644) for oral PrEP. Persistence was 79.3% (242/305) among oral PrEP users, the majority of whom were MSM (78.5% [190/242]) and Black or *Pardo* (73.3% [175/242]). Among LAI-PrEP users, persistence was 88.8% (301/339), and the majority were likewise MSM (80.7% [243/301]) and Black or *Pardo* (74.1% [223/301]) (Fig. 3).

In bivariate analyses, initiation of LAI-PrEP was significantly higher when compared to oral PrEP initiation among participants from São Paulo (48.7% vs. 38.6%) and Belo Horizonte (25.8% vs. 17.5%) (p < 0.001), among prior PrEP users (35.5% vs. 21.4%; p < 0.001), and among those who reported being discriminated against based on gender identity or sexual orientation (52.2% vs. 40.3%; p = 0.003). Among those who initiated oral PrEP, switching to LAI-PrEP was significantly higher among Black or *Pardo* SGDA (82.2% vs. 71.6%; p-value = 0.046) and those with elementary or high school education (79.8% vs. 68.6%; p-value = 0.043) (Table 1).

In the final adjusted regression model, participants with prior experience with oral PrEP had higher odds of initiating LAI-PrEP compared to those who did not use it before (aOR 2.07; 95% CI: 1.35–3.17), or had experienced discrimination based on gender identity or sexual orientation in the past 6 months (aOR 1.69; 95% CI: 1.19–2.41). Black or *Pardo* SGDA had higher odds of switching from oral PrEP to LAI-PrEP than White SGDA (aOR 2.03; 95% CI: 1.03–4.02) (Table 4). Both sensitivity models yielded aORs in the same direction with overlapping CIs (Supplementary Table S3).

**Table 4:** Final multivariate model of factors associated with LAI-PrEP initiation and switching to LAI-PrEP among SGDA. PrEP15-19 Choices Study, April/2024–September/2025.

Variables	LAI-PrEP initiation^a^ (n = 567)		Switching to LAI-PrEP^b^ (n = 316)	
	aOR	95%CI	aOR	95%CI
Race/skin color				
White			1.00	–
Black/*Pardo*			2.03	1.03–4.02
Prior oral PrEP use				
No	1.00	–		
Yes	2.07	1.35-3.17		
Discrimination based on gender identity or sexual orientation in the last 6 months				
No	1.00	–		
Yes	1.69	1.19-2.41		

PrEP, pre-exposure prophylaxis; LAI-PrEP, long-acting injectable pre-exposure prophylaxis.

a Oral PrEP (i.e., both daily and event-driven regimens) initiation vs. LAI-PrEP initiation model adjusted for skin color, timeframe of sexual engagement, and random effects by site.

b Continued oral PrEP (i.e., both daily and event-driven regimens) vs. switched to the LAI-PrEP model adjusted for schooling, income, and prior oral PrEP use.

## Discussion

This study investigated PrEP uptake across three modalities and explored patterns of choice dynamics among SGDA aged 15–19 years in a real-world setting. LAI-PrEP attracted more than one third of adolescents who had not previously used oral PrEP (38.5%; [180/467]), suggesting that injectable options are not merely alternatives for those facing adherence challenges but are essential for expanding coverage among populations historically underserved by oral regimens. Furthermore, participants who initiated PrEP using oral formulations were associated with higher odds of transition to LAI-PrEP. For some adolescents, starting HIV prevention with oral PrEP may serve as an initial step towards adopting injectable formulations, driven by improved accessibility, strong confidence in the regimen, and the belief that it better aligns with their sexual behavior and lifestyle. These findings highlight the importance of offering a range of PrEP modalities to support personalized HIV-prevention strategies, particularly among diverse adolescent populations with varying needs and preferences.^20^

In our study, PrEP uptake was 30.0% (644/2150), marking a notable improvement compared to the earlier PrEP15-19 study at the same sites conducted by our research group. This uptake was almost double that reported by Magno et al.,^21^ who found a 15.8% uptake (estimated by the authors based on the number of adolescents included in PrEP by the total of adolescents reached by DCS [716/4529]). This increase may be related to individuals with prior experience with PrEP. However, the effect of an expanded range of PrEP options that are now available must also be considered, as this may appeal to adolescents who might otherwise remain disengaged from PrEP services. Additionally, this higher uptake may be influenced by broader eligibility criteria, recent national guideline changes in Brazil extending PrEP access to individuals aged 15–17 years old,^8^ and enhanced public awareness campaigns.

Despite previous research indicating a strong initial interest in LAI-PrEP among Brazilian adolescents,^20^ our findings revealed lower expected rates of initiation, with just over 40% of the participants initially choosing this modality. Notably, even in studies reporting high initial interest, the uptake of LAI-PrEP tends to decline with younger age. Moreover, much of the existing evidence comes from studies conducted on adult populations in the United States, Kenya, Uganda, where higher initiation rates have been observed.^22^^,^^23^ Differences in demographic characteristics, particularly age and gender identity, may partly account for these disparities, as previous studies have primarily focused on older cisgender women who face distinct barriers and facilitators compared to SGDA populations. In Brazil, awareness of LAI-PrEP remains limited among SGDA, and this option has not yet been integrated into the services of the Brazilian National Health System (in Portuguese: *Sistema Único de Saúde–SUS*). As a result, low familiarity with the method and limited peer- or community-level dissemination may have reduced participants’ confidence in initiating injectable PrEP. This unfamiliarity likely contributed to a preference for beginning oral PrEP, with some individuals later transitioning to LAI-PrEP as they became more comfortable and confident in its use.

We observed a notable divergence between adolescents’ initial self-reported willingness to start using injectable methods and their actual initiation rates after clinical consultations. This shift underscores the significant influence of clinical counseling, thorough assessments, and adolescents’ subsequent re-evaluation of their readiness to commit to an injectable regimen. This pattern suggests that some adolescents may view oral PrEP as a preliminary step towards LAI-PrEP, using an oral regimen to evaluate their comfort and readiness before committing to an injectable option. By the end of the follow-up period, the proportion of adolescents using LAI-PrEP closely mirrored the initial intention to use it reported at recruitment.

Our analysis also identified specific factors associated with LAI-PrEP initiation. Adolescents with prior experience using oral PrEP and those who reported discrimination based on gender identity or sexual orientation in the past 6 months were significantly associated with higher odds of initiating LAI-PrEP. Additionally, Black and *Pardo* SGDA had higher odds of switching from oral PrEP to LAI-PrEP than their peers who remained on oral PrEP. Our findings provide two key insights. First, adolescents’ choice of PrEP modality appears to be shaped not only by the feasibility of its use but also by prior experience with prevention strategies. Second, for socially marginalized adolescents, particularly SGDA, who experience discrimination or belong to racially vulnerable groups, injectable PrEP may be a way of facing the stigma and structural vulnerability they suffer, highlighting that these aspects limit the possibility of making a free choice. It is important to recognize that oral PrEP poses unique challenges for adolescents, including difficulties in storing pill bottles discreetly,^24^ which LAI-PrEP may help to mitigate. Methods for mitigating these situations must be considered during the PrEP offering.

Additional obstacles include incorporating daily medication into their routines and managing social consequences, such as HIV-related stigma and fear of discrimination.^25^^,^^26^ LAI-PrEP offers a promising alternative that may reduce these burdens and improve adherence among youth. In addition to its role as an HIV-prevention tool, PrEP has been framed as a means of promoting sexual empowerment.^27^ Moreover, it may help mitigate the stigma associated with daily HIV medication and provide greater freedom in sexual decision-making.^28^^,^^29^ Therefore, promoting a decision-making process that enhances adolescents’ knowledge and sense of safety regarding injectable PrEP can help ensure that their choices align more closely with their individual needs and circumstances.

Our results highlight that adolescents’ choice of PrEP modality is often driven more by their experience with HIV prevention than by the realities of effective use. For socially marginalized adolescents, LAI-PrEP is frequently viewed as a strategy for mitigating existing structural barriers. Oral PrEP poses several well-documented challenges for youth, including concerns about medication storage,^21^ difficulty in incorporating a daily pill into their routine, and managing stigma and fear of discrimination.^23^^,^^24^ LAI-PrEP offers a promising alternative to reduce these burdens and potentially improve adherence among youth. Therefore, promoting a selection process that increases informed choice and supports the safe use of injectable medications is crucial for meeting their needs.

Despite the high initial willingness to use LAI-PrEP, a notable gap between initial willingness and actual initiation was observed, reflecting patterns in which structural and clinical barriers limit its uptake in other settings. Even in this no-cost study context, factors such as eligibility criteria, clinical delays (e.g., pending PEP completion), and personal hesitation likely contributed to delayed or foregone initiation. Additionally, 19 suicidality events were recorded among adolescents who initiated LAI-PrEP, which led us to adopt mental health criteria for not prescribing injectable PrEP to those at risk of suicide, which was followed by a reduction in the incidence of these cases.

Strengthening clinical protocols, implementing procedures to ensure timely linkage during the initiation period, and integrating mental health support to help adolescents navigate psychosocial and structural barriers may help reduce the gaps in PrEP decision-making and initiation. Future studies should be designed to deepen our understanding of this issue as well as to improve clinical protocols, implementing procedures to ensure the timely identification of cases that need care and integrating PrEP services and mental health support to help adolescents navigate the psychosocial and structural situations they are subject to.

This study has limitations. First, convenience sampling may limit the generalizability of our findings. Although the study included longitudinal follow-up, most covariates were measured at enrollment. This may have limited our ability to fully capture changes in participants’ characteristics and PrEP use patterns over time. Thus, the variables of switching and persistence may have been underestimated because of the unbalanced follow-up of the participants in this study. Furthermore, the estimated uptake presented in this analysis included only adolescents who were recruited by peer educators, expressed an interest in learning about or using PrEP, and provided the minimum information required for enrollment. However, the total number of contacts made by peer educators and the broader scope of outreach during recruitment were substantially larger, suggesting that our estimates represent only a subset of the broader target population. Modality access in our setting was structurally constrained by provider role, which likely influenced modality choice. Although our models included a site/service random intercept, residual confounding by provider role and care model may remain.

Despite these limitations, our findings underscore the critical importance of offering diverse HIV prevention options tailored to the needs of SGDA. Although oral PrEP was initially preferred by most Brazilian adolescents, the significant proportion of adolescents who later transitioned to injectable LAI-PrEP highlights the importance of providing multiple PrEP modalities. Future initiatives should prioritize addressing adolescents’ concerns about injectable formulations while increasing awareness of their unique benefits. Integrating accessible mental health programs and psychosocial support into PrEP delivery will be essential. Such targeted efforts have the potential to enhance adherence rates and improve HIV-prevention outcomes in this vulnerable population.

## Contributors

ID, AG, and LM conceptualized the study, oversaw all phases of implementation, and provided overall coordination. ID, AG, and DG secured funding. FS, LD, and PM supervised data collection and site-level coordination. BOL performed the statistical analysis. MW, PM, and UT supported data curation and interpretation. All authors contributed to the study design, ethical oversight, and critical revision of the manuscript. LM drafted the initial manuscript, with substantial input from DZ, BOL, ID, MW, and LD. All authors reviewed and approved the final version and accepted responsibility for the decision to submit the manuscript for publication.

## Data sharing statement

The individual participant data that underline the results reported in this article, including data tables, relevant figures, and a data dictionary defining each variable, will be made available to researchers whose proposed use of the data has been approved by the study investigators. Data will be made available for publication and will remain accessible for 5 years. The data will be stored in a secure institutional data repository maintained by the *Universidade do Estado da Bahia* (UNEB). Access can be obtained by contacting the corresponding author upon request for data access, which will be reviewed by the study’s data access committee. The researchers were required to submit a brief proposal outlining the intended use of the data. Access will be granted for non-commercial academic research purposes only, subject to the signing of a data access agreement and compliance with ethical standards. No support for data analysis will be provided and no identifying information will be shared. Where applicable, the statistical code used in the analysis will also be shared upon request.

## Disclosure of AI or AI-assisted technologies

English review was provided by ChatGPT (OpenAI) under the direction and supervision of the authors. This support was limited to language editing and did not influence the interpretation of results or conclusions.

## Declaration of interests

We declare no competing interests.
