# Supplementary material for: PrEP uptake, persistence, and patterns of choice dynamics between oral and long-acting injectable with cabotegravir among sexually and gender-diverse adolescents in Brazil: a multisite cohort implementation study
Source: Lancet Reg Health Am. 2026 May 28;59:101515. doi: 10.1016/j.lana.2026.101515 (PMC13235330; doi:10.1016/j.lana.2026.101515)
Supplement: Appendix 1 and Supplementary Tables S1–S3 [file mmc1.pdf]

Supplementary material

PrEP uptake, persistence, and patterns of choice dynamics between oral and long-acting injectable with cabotegravir among sexually and gender-diverse adolescents in Brazil: a multisite cohort implementation study

Laio Magno[1,2,3,*], Beo Oliveira Leite[1,3], Diana Zeballos[1,3], Fabiane Soares[1, 3], Lorenza Dezanet[4,5], Mateus Westin[4], Unaí Tupinambás[4], Dirceu Greco[4], Paula Massa[6,7], Alexandre Grangeiro[6], Inês Dourado[3], on behalf of the PrEP15-19 Choices Study[‡]

Content

Appendix 1: PrEP15-19 Choices Study Group

Table S1: Characteristics of SGDA participants included and not-included in the PrEP15-19 Choices Study, April/2024-September/2025 (N=2,150).

Table S2: Reasons for switching from LAI-PrEP to Oral PrEP in the PrEP15-19 Choices Study, April/2024-September/2025 (n=30)

Table S3: Sensitivity analysis of the final multivariate model of factors associated with LAI-PrEP initiation versus oral PrEP initiation among SGDA. PrEP15-19 Choices Study, April/2024–September/2025

Appendix 1: PrEP15-19 Choices Study Group:

First and middle names	Surnames	Affiliations
Thais Regis Aranha	Rossi	Universidade do Estado da Bahia
Priscilla	Caires	Universidade Federal da Bahia
Filipe Mateus	Duarte	Universidade Federal da Bahia
Vinícius Damasceno	Nascimento	Universidade Federal da Bahia
Manuela Cunha	Gomes	Universidade Federal da Bahia
Márcia Thereza	Couto	Faculdade de Medicina Universidade de São Paulo
Luiz Felipe Alves de	Sousa	Faculdade de Medicina Universidade de São Paulo
Carolina Cardona Siqueira	Lobo	Faculdade de Medicina Universidade de São Paulo
Eliana Miura	Zucchi	Programa de Pós-Graduação em Saúde Coletiva, Universidade Católica de Santos, Faculdade Israelita de Ciências da Saúde Albert Einsten
Eliane Aparecida	Sala	Faculdade de Medicina da Universidade de São Paulo
Ana Paula	Silva	Universidade Federal de Minas Gerais
Marília	Greco	Universidade Federal de Minas Gerais
Patrícia	Hafarrany	Centro de Referência da Juventudes, PBH Belo Horizonte

Table S1. Characteristics of SGDA participants included and not-included in the PrEP15-19 Choices Study, April/2024-September/2025 (N=2,150).

**Variables**	**Not included**<br>**1,506 (70%)**	**Included**<br>**644(30%)**	**p-value***
Age			0·492
15-17 years old	1,109 (74%)	465 (72%)	
18-19 years old	397 (26%)	179 (28%)	
Study population			<0·001
MSM	1,261 (84%)	506 (79%)	
TGW	170 (11%)	76 (12%)	
TGM	28 (2%)	12 (2%)	
NB	47 (3%)	50 (4%)	
Race/Skin color			<0·001
White	623 (44%)	171 (27%)	
Pardo	401 (28%)	224 (35%)	
Black	404 (28%)	248 (39%)	

*Pearson's chi-squared; SGDA, sexually and gender-diverse adolescents; PrEP, pre-exposure prophylaxis; MSM, men who have sex with men; TGW, transgender women; TGM, transgender men; NB, non-binary.

Table S2. Reasons for switching from LAI-PrEP to Oral PrEP in the PrEP15-19 Choices Study, April/2024-September/2025 (n=30)

**Site**	**Gender identity**	**Race/skin color**	**Age***	**Reason**
Salvador	Cisgender man	*Pardo*	17	Mental health criteria
Salvador	Cisgender man	*Pardo*	18	Mental health criteria
Salvador	Cisgender man	*Pardo*	16	Pain at injection site
Salvador	Cisgender man	Black	18	Fever, Headache and Pain at injection site
Salvador	Cisgender man	*Pardo*	20	Mental health criteria
São Paulo	Cisgender man	White	20	Distance from home to health clinic
São Paulo	Non-binary assigned male at birth	White	21	Mental health criteria
São Paulo	Transgender woman	White	19	Mental health criteria
São Paulo	Transgender woman	Black	20	On behalf of the job
São Paulo	Cisgender man	*Pardo*	19	Pain at injection site
São Paulo	Cisgender man	*Pardo*	20	Mental health criteria
São Paulo	Cisgender man	*Pardo*	20	No reason related
São Paulo	Cisgender man	Black	22	Pain at injection site
São Paulo	Non-binary assigned male at birth	Black	21	Pain at injection site
São Paulo	Transgender woman	Black	22	No reason related
São Paulo	Transgender woman	Indigenous	20	No sexual intercourse
São Paulo	Transgender woman	*Pardo*	20	Mental health criteria
São Paulo	Transgender woman	White	20	No reason related

São Paulo	Cisgender man	*Pardo*	19	Pain at injection site
São Paulo	Non-binary assigned male at birth	White	18	Pain at injection site
São Paulo	Cisgender man	White	19	Pain at injection site
São Paulo	Cisgender man	White	19	Pain at injection site
Belo Horizonte	Transgender woman	Black	16	Mental health criteria
Belo Horizonte	Transgender woman	Black	20	Mental health criteria
Belo Horizonte	Cisgender man	*Pardo*	20	Mental health criteria
Belo Horizonte	Cisgender man	White	18	No reason related
Belo Horizonte	Cisgender man	*Pardo*	18	Mental health criteria
Belo Horizonte	Cisgender man	Black	19	No reason related
Belo Horizonte	Cisgender man	*Pardo*	18	Mental health criteria
Belo Horizonte	Cisgender man	White	19	No sexual intercourse

* Age refers to age at the time of PrEP modality change, not age at enrollment; therefore, some participants were older than 19 years at the event despite meeting eligibility criteria at study entry.

Table S3: Sensitivity analysis of the final multivariate model of factors associated with LAI-PrEP initiation versus oral PrEP initiation among SGDA. PrEP15-19 Choices Study, April/2024–September/2025

Variables	Model 1[1] (n=546) LAI-PrEP initiation[3]		Model 2[2] (n=567) LAI-PrEP initiation[3]	
	aOR	95%CI	aOR	95%CI
**Prior oral PrEP use**				
No	1·00	-	1·00	-
Yes	1·99	1·29-3·07	1·90	1·24-2·91
**Discrimination based on gender or sexual orientation in the last 6 months**				
No	1·00	-	1·00	-
Yes	1·76	1·23-2·51	1·75	1·23-2·48
**Variables**	**Model 2 (n=310) Switching to LAI-PrEP[4]**		**Model 2 (n=316) Switching to LAI-PrEP[4]**	
	aOR	95%CI	aOR	95%CI
**Race/skin color**				
White	1·00	-	1·00	-
Black/*Pardo*	1·90	0·95-3·78	2·04	1·05-3.95
**Prior oral PrEP use**				
No	1·00	-	1·00	-
Yes	2·00	1·00-4·3	2·07	1·07-4·01

[1]Model 1 – excludes participants who expressed willingness to use LAI-PrEP but were ineligible;

[2]Model 2 classified participants who were ineligible for LAI-PrEP based on mental health criteria as LAI-PrEP users if they mentioned their willingness to use LAI-PrEP.

[3]Oral PrEP initiation (both daily and on-demand regimens) vs. LAI-PrEP initiation model adjusted for skin color, timeframe of sexual engagement, and random effects by site.

[4]Kept in oral PrEP vs. switched to LAI-PrEP model adjusted by schooling, income and prior oral PrEP use;

PrEP, pre-exposure prophylaxis; LAI-PrEP, long-acting injectable pre-exposure prophylaxis.
